# Right thoracic curvature in the normal spine

**DOI:** 10.1186/1749-799X-6-4

**Published:** 2011-01-14

**Authors:** Toshio Doi, Katsumi Harimaya, Hiromichi Mitsuyasu, Yoshihiro Matsumoto, Keigo Masuda, Kazu Kobayakawa, Yukihide Iwamoto

**Affiliations:** 1 Department of Orthopaedic Surgery, Graduate School of Medical Sciences, Kyushu University, Fukuoka, Japan

## Abstract

**Background:**

Trunk asymmetry and vertebral rotation, at times observed in the normal spine, resemble the characteristics of adolescent idiopathic scoliosis (AIS). Right thoracic curvature has also been reported in the normal spine. If it is determined that the features of right thoracic side curvature in the normal spine are the same as those observed in AIS, these findings might provide a basis for elucidating the etiology of this condition. For this reason, we investigated right thoracic curvature in the normal spine.

**Methods:**

For normal spinal measurements, 1,200 patients who underwent a posteroanterior chest radiographs were evaluated. These consisted of 400 children (ages 4-9), 400 adolescents (ages 10-19) and 400 adults (ages 20-29), with each group comprised of both genders. The exclusion criteria were obvious chest and spinal diseases. As side curvature is minimal in normal spines and the range at which curvature is measured is difficult to ascertain, first the typical curvature range in scoliosis patients was determined and then the Cobb angle in normal spines was measured using the same range as the scoliosis curve, from T5 to T12. Right thoracic curvature was given a positive value. The curve pattern was organized in each collective three groups: neutral (from -1 degree to 1 degree), right (> +1 degree), and left (< -1 degree).

**Results:**

In child group, Cobb angle in left was 120, in neutral was 125 and in right was 155. In adolescent group, Cobb angle in left was 70, in neutral was 114 and in right was 216. In adult group, Cobb angle in left was 46, in neutral was 102 and in right was 252. The curvature pattern shifts to the right side in the adolescent group (p < 0.01) and in adult group (p < 0.001) compared to the child group. There was no significant difference in curvature pattern between adolescent and adult group.

**Conclusions:**

Based on standing chest radiographic measurements, a right thoracic curvature was observed in normal spines after adolescence.

## Background

Coronal side curvature deformity, trunk asymmetry, and spinal body rotation are observed in patients with adolescent idiopathic scoliosis (AIS). Many studies have been conducted to elucidate the etiology of scoliosis [1,2]. In spite of these numerous investigations, it is still unclear why AIS begins in adolescence and why right thoracic scoliosis is more common than left thoracic scoliosis.

Trunk asymmetry [1,3,4], right thoracic vertebral rotation [5], and right thoracic curvature have been reported in the normal spine [6,7]. Trunk asymmetry is prominent after adolescence [8]. The above characteristics resemble the deformities observed in AIS. The prevalence of scoliosis curves with angles greater than 10 degrees is reported to range from 0.5% to 3% [9-11]. This curvature data is based on the spines of students undergoing scoliosis screening, however, there is no information on the distribution of thoracic side curvature in the normal spine. Moreover, there is no data on whether or not thoracic side curvature changes during growth. In this study, we studied thoracic curvatures using standing chest radiographs of children, adolescents, and adult patients who came to our hospital with no obvious chest or spinal diseases.

## Methods

### Subjects

#### Scoliosis Patients

To study the extent of thoracic scoliosis deformities, we evaluated 44 consecutive patients without congenital and symptomatic scoliosis who were seen at our hospital between January 2008 and December 2008. For these patients, Cobb angles ranged from 15 to 75 degrees (average 39.1), ages ranged from 5 to 19 years old (average 12.7), and 2 were male and 42 were female.

#### Normal spinal measurements

We recruited a standing posteroanterior chest radiographs on 1,200 patients, who were seen at our hospital, from January 2008 to August 2008. Patients with a scoliosis curve of over 10 degrees and obvious chest and spine disease seen in radiograph were excluded. We evaluated three groups of patients: children (ages 4-9), adolescents (ages 10-19), and adults (ages 20-29). Two hundred consecutive individuals, both male and female, were measured for each group (total 1200 patients).

### Radiological methods

The degree of curvature was assessed with the Cobb method [12]. For the measurement of the Cobb angle in scoliosis patients, standing AP radiographs were undertaken using a long-cassette radiographs of the spine. Radiographs were transferred on computer screen by Fuji Synapse System (FujiFilm holdings, Tokyo, Japan) and the upper and lower end vertebrae were determined, and degrees of two lines along with each end vertebrae were calculated by the angle measurer (Fuji Synapse System). For the measurement of the Cobb angle in 1,200 normal spines, standing chest PA radiographs were obtained. On the computer screen using Fuji Synapse angle measurer, a line is drawn along the superior end plate of T5 and a second line drawn along the lower end plate of T12. If the end plate was indistinct the line was drawn through the pedicles. A right convex curve was assigned a positive value, and a left curve a negative value. The curve pattern was organized in each collective three groups: neutral (from -1 degree to 1 degree), right (> +1 degree), and left (< -1 degree). The curvature pattern difference in each generation was analyzed by Kruskal-Wallis test with post-hoc test.

### Intra-observer and inter-observer reliability

Three orthopaedic surgeons (observer 1, observer 2, and observer 3) were familiarized with the computer program and also taught how to place the vertebral landmarks on the computer monitor. The measurements were carried out twice on different occasions with 12 radiographs. The intervals between measurements were at least 2 weeks. Intraobserver and interobserver agreement was assessed by the interclass correlation coefficient.

Prism statistical software was used for statistical analysis. Statistical methods included the Kruskal-Wallis test.

## Results

### Thoracic curvature in scoliosis patient

**Figure 1:**
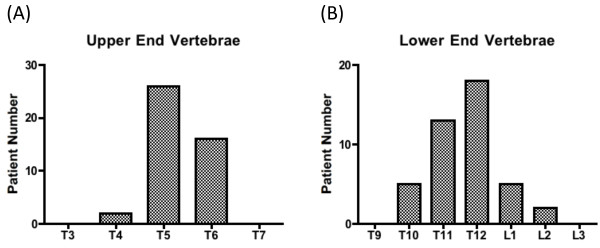
**Range of curvature in scoliosis patients**. The upper and lower end vertebrae were examined in scoliosis patients. The median of the upper end was at T5 and the lower end at T12. All were instances of right thoracic scoliosis.

We assessed the side curve patterns of scoliosis patients before measuring normal spines. The upper and lower end vertebrae were determined for AIS patients. All scoliosis patients had a right thoracic curvature, with an upper end vertebra of T5.3 ± 0.6, a lower end vertebra was T11.7 ± 1.0, and an apex of T8.5 ± 0.8, by anatomical levels were averaged over the patients (Figure 1). The medians were T5 and T12 for the upper and lower end vertebrae, respectively.

### Thoracic right curvature in the normal spine

To evaluate the curvature in the normal spine, Cobb angles from T5 to T12 were measured using a standing chest radiographs. As it is difficult to identify vertebral bodies on standing chest Radiographs, the contrast was changed to facilitate recognition.

The interclass correlation coefficient for the intraobserver reliability of the Cobb angle measurements using chest radiograph was 0.82 for observer 1, 0.61 for observer 2, and 0.79 for observer 3 (Table 1).

**Table 1:** Intraobserver reliability analysis for the measurement of Cobb angle.

	Interclass correlation coefficient
Observer 1 (N = 12)	0.81 (0.67-0.90)

Observer 2 (N = 12)	0.61 (0.36-0.78)

Observer 3 (N = 12)	0.79 (0.63-0.88)

Each observer measured CT images (N = 12) and interclass correlation coefficient was calculated. Numerical value shows the gamma of interclass correlation coefficient and the range of the 95% confidence interval.

The interclass correlation coefficient for the interobserver reliability of the Cobb angle measurements using chest radiographs by the three observers was 0.70 (Table 2).

**Table 2:** Intrerobserver reliability analysis for the measurement of Cobb angle.

	Interclass correlation coefficient	95% confidence interval
Cobb angle measuremet (N = 36)	0.70	0.52-0.82

Each observer measured CT images (N = 36) twice on different occasions and the interclass correlation coefficient was calculated.

**Figure 2:**
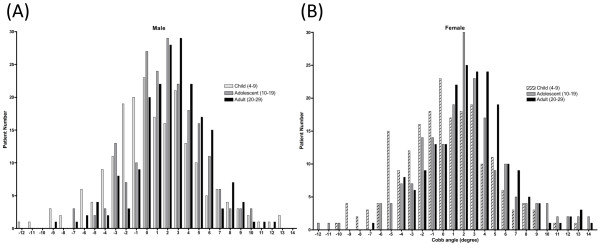
**Extent of side curvature in the normal spine**. The degree of the side curvature in children (ages 4-9), adolescents (ages 10-19), and adults (ages 20-29) is shown. Right-sided curvature is given a positive value. (A) shows male and (B) shows female.

**Figure 3:**
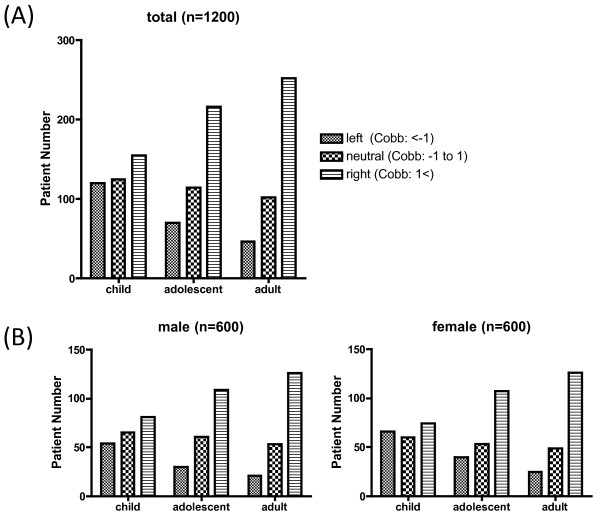
**Right thoracic curvature is prominent after adolescence**. (A) The side curvature was organized in collective three groups: neutral (from -1 degree to 1 degree), right (> +1 degree) and left (< -1 degree). The curvature pattern shifts to the right side in the adolescent group (p < 0.01) and in the adult group (p < 0.001) compared to the child group. (B) There was no significant difference in the curvature pattern between male and female.

The Cobb angles for each group are shown in Figure 2. In children, the Cobb angle was 0.6 ± 3.7 degrees (mean ± SD) in males and 0.1 ± 3.9 degree in females. In adolescents, the Cobb angle was 1.8 ± 2.2 degrees in males and 1.5 ± 3.3 in females. In adults, the Cobb angle was 2.3 ± 3.2 degrees in males and 2.3 ± 3.1 degrees in females. The side curvature was organized in collective three groups: neutral (from -1 degree to 1 degree), right (> +1 degree) and left (< -1 degree). In child group, Cobb angle in left was 120, in neutral was 125 and in right was 155. In adolescent group, Cobb angle in left was 70, in neutral was 114 and in right was 216. In adult group, Cobb angle in left was 46, in neutral was 102 and in right was 252 (Figure 3A). The curvature pattern shift to the right side in the adolescent group (p < 0.01) and in the adult group (p < 0.001) compared to the child group in both genders by Kruskal-Wallis test with post-hoc test. There was no significant difference in curvature pattern between adolescent and adult group. There was no significantly difference in the curvature pattern between male and female in adolescent and adult group (Figure 3B).

## Discussion

Right thoracic scoliosis, trunk asymmetry, and thoracic vertebral right rotation are among the characteristics of AIS. Even in the normal spine, trunk asymmetry [1] and thoracic vertebral right rotation [4,5] have been reported. Trunk asymmetry may become prominent in the normal spine after adolescence [8]. Right thoracic curvature has also been reported in the normal spine [6,7]. Much data on the prevalence of scoliosis are based on school screening examinations, scapular prominence, asymmetric shoulder levels, and rib humps observed during the forward-bending test [10,11,13]. Side curvature has been detected in about 2% of school children using scoliosis screening. Only individuals suspected of having scoliosis undergo a radiograph, and therefore the distribution patterns of side curvature and the average curves of the normal spine are unknown. To determine the distribution pattern of thoracic curvature in the normal spine, we measured the curvature using standing chest radiographs in the normal spines of children, adolescents, and adults.

In AIS, curvature is prominent during adolescence and worsens during growth spurts. Interestingly, right thoracic curvature was also observed in normal spines and was prominent after adolescence (Figure 3), thus resembling the pattern observed in AIS.

The ratio of boys to girls impacted by AIS is equal for minor curves, yet as the magnitude of the curvature increases more girls are affected, with the ratio reaching 1:8 for those requiring treatment [9,10,14]. Our study suggests that the degree of right thoracic curvature in the normal spine is the same in males and females.

There are inherent limitations of the study - the 1200 subjects recruited are not true 'normal controls' randomly selected but were actually recruited from subjects attending hospital due to other medical condition or for check up. For normal spinal observations, we used patients without obvious chest and spinal diseases as determined by chest radiographs because the literature suggests that patients who have congenital heart disease are more likely to have scoliosis [15-17]. It is possible, however, that our study population had heart and lung diseases that were not indicated on the chest radiographs, and it would therefore be ideal to recruit normal volunteer. However, it is difficult to require healthy subjects to undergo radiographic examinations because of the unnecessary exposure to radiation and the ethical problems. The other limitation of the study is the lack of sagittal profile information of the spine. It is useful to examine the CT scan for the analysis of sagittal curvature, however, it is also difficult to require healthy subjects to undergo CT scans by obvious ethical reasons.

In addition to trunk asymmetry and rightward vertebral body rotation in the normal spine, we demonstrated the presence of right thoracic curvature. These deformities have the same features as AIS. Moreover, they become prominent after adolescence, which follows the same trend observed in AIS. These findings support the possibility that the worsening of deformities existing in normal individuals is the mechanism of AIS progression. An as yet unidentified factor (or factors) may exist that evokes a right thoracic curvature in the normal spine, for example heart location as shown by a study of dextrocardia [18], and when it worsens AIS may occur. Further studies should be conducted to examine in more detail the mechanism in which deformities in the normal spine are involved in the etiology of AIS.

## Conclusions

Measurements of standing chest radiographs were used to study the thoracic side curvature in normal spines. A significant right thoracic curvature in the normal spine was observed after adolescence.

## Competing interests

The authors declare that they have no competing interests.

## Authors' contributions

TD has contributed to conception and design of the study, acquisition of data, analysis and interpretation of data, and drafting the manuscript. KH, HM, and YM performed part of literature review. KM and KK performed part of acquisition of data. YI participated in design and coordination and helped to draft the manuscript. All authors read and approved the final manuscript.
